# POLICY PRIORITIES: PERSPECTIVES FROM THE BEHAVIORAL AND SOCIAL SCIENCES

**DOI:** 10.1093/geroni/igz038.1456

**Published:** 2019-11-08

**Authors:** Karl Pillemer

**Affiliations:** Cornell University, Ithaca, New York, United States

## Abstract

This presentation will cover public policy issues of significance to the aging population, focusing on the perspective of the behavioral and social sciences and on policies that may improve physical and mental health.

